# Effectiveness and Cost-Effectiveness of a Cluster-Randomized Prenatal Lifestyle Counseling Trial: A Seven-Year Follow-Up

**DOI:** 10.1371/journal.pone.0167759

**Published:** 2016-12-09

**Authors:** Päivi Kolu, Jani Raitanen, Jatta Puhkala, Pipsa Tuominen, Pauliina Husu, Riitta Luoto

**Affiliations:** 1 UKK Institute for Health Promotion, Tampere, Finland; 2 School of Health Sciences, University of Tampere, Tampere, Finland; Jichi Medical University, JAPAN

## Abstract

There is a link between the pregnancy and its long-term influence on health and susceptibility to future chronic disease both in mother and offspring. The objective was to determine whether individual counseling on physical activity and diet and weight gain at five antenatal visits can prevent type 2 diabetes mellitus (T2DM) and overweight or improve glycemic parameters, among all at-risk-mothers and their children. Another objective was to evaluate whether gestational lifestyle intervention was cost-effective as measured with mother’s sickness absence and quality-adjusted life years (QALY). This study was a seven-year follow-up study for women, who were enrolled to the antenatal cluster-randomized controlled trial (RCT). Analysis of the outcome included all women whose outcome was available, in addition with subgroup analysis including women adherent to all lifestyle aims. A total of 173 women with their children participated to the study, representing 43% (173/399) of the women who finished the original RCT. Main outcome measures were: T2DM based on medication use or fasting blood glucose or oral glucose tolerance test (OGTT), body mass index (BMI), glycosylated hemoglobin (HbA1c). None of the women were diagnosed to have T2DM. HbA1c or fasting blood glucose differences were not found among mothers or children. Differences in BMI were non-significant among mothers (Intervention 27.3, Usual care 28.1 kg/m2, p = 0.33) and children (I 21.3 vs U 22.5 kg/m2, p = 0.07). Children’s BMI was significantly lower among adherent group (I 20.5 vs U 22.5, p = 0.04). The mean total cost per person was 30.6% lower in the intervention group than in the usual care group (I €2,944 vs. U €4,243; p = 0.74). Intervention was cost-effective in terms of sickness absence but not in QALY gained i.e. if society is willing to pay additional €100 per one avoided sickness absence day; there is a 90% probability of the intervention arm to be cost-effective. Long-term effectiveness of antenatal lifestyle counseling was not shown, in spite of possible effect on children’s BMI. Cost-effectiveness of the intervention in terms of sickness absence may have larger societal impact.

## Introduction

There is a link between the pregnancy and its long-term influence on health and susceptibility to future chronic disease both in mother and offspring [1]. Inactivity and unhealthy eating habits during pregnancy increase risk for prenatal excess weight gain and increase risk for type 2 diabetes mellitus (T2DM) and cardiovascular diseases later in life [2, 3]. Additionally, maternal obesity may predispose children for impaired glucose tolerance, [4] and increase also risk for childhood obesity [5–7].

The importance of maternal lifestyle counseling is obvious, because globally 37.3% of adult women are overweight or obese [8] and 26.8% are inactive (i.e. not meet the physical activity recommendation) [9]. In Finland the situation is even worse, because 48.6% of adult women are at least overweight (body mass index ≥ 25) and 28.1% are inactive [8, 9]. Pregnancy and postpartum period may predispose women to healthy lifestyle and prevent future chronic diseases. Consequently both individualized dietary counseling and support to at least moderate intensity physical activity or combination of them has shown to be restrain gestational weight gain on healthy pregnant women [10, 11] but the effect of diet and exercise interventions on gestational diabetes mellitus (GDM) incidence is limited [12, 13]. We have previously reported favorable changes in diet composition and proportion of large-for-gestational-age newborns in a cluster-randomized trial in order to prevent GDM [14–17]. However, long-term effectiveness of lifestyle counseling interventions among women with risk of GDM or their children have not yet been published in our or any other trial.

Short-term cost-effectiveness of our own lifestyle counseling intervention among GDM risk group was not favorable for birthweight, quality of life (15D) or perceived health [18]. Parallel negative cost-effectiveness findings to our trial were reported in Oostdam et al. [19] study, where maternal fasting blood glucose, insulin sensitivity or quality-adjusted life year (QALY) were used as outcome indicators.

Objective for this study was to determine whether lifestyle counseling during pregnancy can prevent T2DM, glucose intolerance or overweight among all at-risk-mothers and their children and for women adherent to all lifestyle aims during gestation. Hypothesis was that T2DM can be prevented in the long term implementing individual physical activity and dietary counseling, because overweight, glucose-intolerance and type 1 or 2 diabetes in first- or second-degree relatives are strong risk factors for T2DM later in life. Another objective was to evaluate whether gestational lifestyle intervention was cost-effective as measured with mother’s sickness absence and quality-adjusted life years.

## Materials and Methods

### Study design and participants

The study was a 7-year follow-up study (mean 7.2, median 7.2, range 5.6–8.3) of the cluster randomized lifestyle counseling trial among women with risk of GDM (n = 399) (trial registration ISRCTN33885819; see http://www.controlled-trials.com/) [16, 20]. For this 7-year-follow-up study were invited all participants in the original intervention who were along at the end of the trial. Women participating to the original trial from the beginning to the end of pregnancy (N = 399) and were willing to participate to the follow-up study (N = 173), were included to the analysis of this article. Response rate to the follow-up study was therefore 43.4% (173/399). The research was conducted in accordance with prevailing ethics principles. Written informed consent was obtained from the mothers and from the guardians on behalf of the children enrolled in study. The informed consent and all questionnaires were recorded. This follow-up study and written informed consent procedure were approved by the medical ethics committees of the Pirkanmaa hospital district (R14039). The follow-up study was performed between May 2014 and January 2016.

#### Original RCT trial

Public health nurses recruited women willing to participate during their first visit to the antenatal clinic. Women who met at least one of the GDM risk factors were included: BMI ≥ 25 kg/m^2^, GDM or a macrosomic newborn (≥ 4500 g) in any earlier pregnancy, any sign of glucose-intolerance, type 1 or 2 diabetes in first- or second-degree relatives, or age ≥ 40 years, were recruited to the trial. The exclusion criteria were type 1 or 2 diabetes before pregnancy, inadequate proficiency in the Finnish language for the study, age < 18 years, twin pregnancy, physical limitations preventing physical activity, and a pathological value in baseline oral glucose-tolerance test (OGTT) at 8–12 or 26–28 weeks’ gestation.

The intervention involved five out of the 11–15 recommended antenatal care visits, which were divided evenly during gestation from 8–12 until 37 weeks. With the intervention group, the public health nurses focused on combined dietary and physical activity counseling, which were based on national physical activity and dietary recommendations, personalised goals, and regular follow-up on targets aiming to restrain gestational weight gain [20]. Women who were slightly physically active and had an uncomplicated pregnancy were encouraged to engage according to physical activity recommendation at least 150 minutes of moderate or 75 minutes vigorous leisure-time activity at least three days a week with the suggested approach being sessions of 10 minutes duration or more [21]. The women at the control maternity clinics received only routine care and no counseling beyond the usual care. However, in Finland routine maternity care includes some dietary and physical activity counseling [16, 20].

#### 7-year follow-up

All participants of the original RCT were invited to 7-year follow-up visit, which consisted of physical measurements (OGTT and blood samples), anthropometric measurements (weight, height and waist circumference) and blood pressure. In addition, children who were born during the intervention were invited to participate with their mothers. Blood sampling concerned both mother and the child in order to analyse fasting blood sugar and glycosylated hemoglobin HbA1c. Women filled also a questionnaire with questions on lifestyle habits, use of health care services, medication, sickness absence, quality of life and workability. The follow-up questionnaire included the same questions as the original RCT, with addition of more details concerning mother’s mental health, psychological well-being, workability, physical activity, sedentary behaviour, children’s diet, physical activity and chronic diseases. Women were contacted up to 3 times using all possible ways (by phone, e-mail and social media) in order to increase participation to the follow-up measurements. Since we did not have information on outcome variable from all women, intention to treat- analysis was not possible to be performed. Analysis of the outcome included all women whose outcome was available, in addition with subgroup analysis including women adherent to all lifestyle aims.

### Outcome measurements

The long-term effectiveness of the lifestyle intervention was evaluated in term of mothers’ prevalence for T2DM. Secondary outcomes were mother’s body mass index (BMI) and quality of life measured using 15D but also children’s BMI, glycosylated hemoglobin (HbA1c) and fasting blood glucose. T2DMwas determined based on at least one of the following criteria: 1) data on use of medication for T2DM, 2) self-reported information on diagnosed T2DM, 3) fasting blood glucose (> 7.0 mmol/l) or 4) Oral glucose tolerance test (OGTT) (> 11.0 mmol/l two-hour) or 5) HbA1c-measurement (≥ 48 mmol/mol; ≥ 6,5%) [22]. Children’s BMI were calculated using BMI for age calculator for children aged 2–20 years [23].

Cost-effectiveness of the intervention seven years after the original RCT was evaluated in terms of sickness absence and QALY gained. Sickness absence was calculated combining all days in sick leave in spite of the reason during last year (mother’s own or children’s sickness). The QALY data were calculated from the standardized 15D questionnaire, which is a validated instrument for measuring health-related quality of life (HRQoL) [24, 25]. The 15D involves 15 separate dimensions: mobility, vision, hearing, breathing, sleeping, eating, speech, excretion, usual activities, mental function, discomfort and symptoms, depression, distress, vitality, and sexual activity. In generation of the overall HRQoL score, the 15 dimensions are covered by a single index number, from 0 (representing the death) to 1 (denoting the best health state) [24]. To calculate QALY gained the difference in index score between 7-year follow-up and the baseline was taken into account and the index was multiplied by the expected lifetime calculated for each woman separately.

Physical activity was assessed by accelerometer (Hookie AM20, Traxmeet Ltd, Espoo, Finland) signal from three orthogonal directions in raw mode with 100 Hz sampling frequency and ±16 g measurement range. The data was analysed in 6’s epoch length. The accelerometer was attached to an elastic belt on the right side of the waist. The collected raw acceleration data was transformed into actual g-units [26, 27]. Women carried accelerometers for one week (7 consecutive days) in order to achieve objective measurement of usual physical activity. To analyses were included only cases who kept the device at least four days per and at least 10 hours a day. Sedentary behaviour (including lying down and sitting) and standing still were defined as activity corresponding less than 1.5 MET, light physical activity 1.5–2.9 MET and moderate-to-vigorous physical activity 3.0 MET and over.

#### Adherent women

Women were defined to be adherent to the recommendations during intervention, if they fulfilled at least three of the five dietary aims and/or their self-reported physical activity exceeded 800 METminutes/weeks at 36–37 weeks’ gestation and their total weight gain did not exceed their BMI-specific limits which provide limits for weight gain during pregnancy. This definition was reported in the original results of the RCT [16].

### Costs

The economic evaluation included the health-care costs to the municipality, costs raised by the patient, medication and productivity costs from the societal perspective. The information on the use of all health care services (visits to a doctor, a nurse, public health nurse, physiotherapist and in-patient days) during the last 12 months, a number of sickness absence days and medication were obtained by questionnaire. The medication cost included both the patient- and the society-borne amount; in 2015 medication costs were 35% contributing by the society, with patient covered the rest of the amount [28,29]. The health care costs were based on average national costs for health care [30]. The unit costs of visits to physicians and to nurses included salary costs and administrative costs but not laboratory expenses. In addition, the unit cost of inpatient hospital days included the daily inpatient-care charge [30].

All costs were calculated for the 12-month period retrospectively. The unit costs, productivity costs, and medication costs were entered at 2015 price level, in euros [31]. Lost productivity was evaluated by means of self-reported information on absence from part- or full-time work via a questionnaire 12 months retrospectively. At questionnaire was separated absence from work according to reason (mother’s own sickness or children’s sickness). However, at cost-effectiveness analysis all sickness days were added up because in spite of the reason they all increase productivity costs. The salary costs were calculated from women’s average national monthly salary scales in 2014 [32] multiplied by 1.3 to encompass related expenses and entered at 2015 price level. The cost calculation assumed 220 workdays a year. The travel expenses and time costs related to the use of health services were not taking into account because this information was not available.

### Statistical analysis

Baseline characteristics, effects and costs were reported as means and standard deviation (SD) or as frequencies and percentages. The difference between the groups was tested with the T-test and Chi-squared test. Children’s fasting blood sugar was only exception, which was tested with Mann-Whitney U-test, because data was not normally distributed. P-values for outcomes were calculated using linear regression and the baseline (gestational week 8–12) was used to standardize the effect.

To evaluate cost-effectiveness, the differences between groups were analysed via a non-parametric bootstrap approach. Cost effectiveness was expressed in terms of incremental cost-effectiveness ratios (ICERs), which indicate the amount of money that is required to decrease days of sickness absence and increase QALYs gained. Health-related quality of life was judged on the basis of the standardised 15D questionnaire. A bootstrap technique with 5,000 replications [33] and cost-effectiveness acceptability curves were used to assess 95% confidence intervals for purposes of analysing the uncertainty around the point estimate of the ICER. The formula for calculation of ICER is

$$ICER\;=(Cost_{Intervention}–\;Cost_{Control})/\;(Effect_{Intervention}–\;Effect_{Control})$$

The confidence intervals (CIs) were calculated by means of a bias-corrected and accelerated (BCa) method proposed by Efron [34]. The BCa interval is given in terms of percentiles of the bootstrap distribution of differences in means, but the percentiles used are chosen after correction for skewness or ‘acceleration’ â and bias [35]. Missing values (costs) were imputed by using mean values of costs. The results were considered to be statistically significant if p < 0.05. Analyses were performed with SPSS (version 22) and Stata (version 12.1) statistics software.

#### Sensitivity analysis

To evaluate the robustness of the findings, we performed sensitivity analysis in two separate ways. Firstly, we standardized the productivity costs between groups, because sickness absence was the highest separate cost. The second sensitivity analysis was a complete case analysis. This entailed including only those participants whose data were available in full, without imputation with respect to direct and productivity cost and the 15D data.

## Results

**Fig 1:**
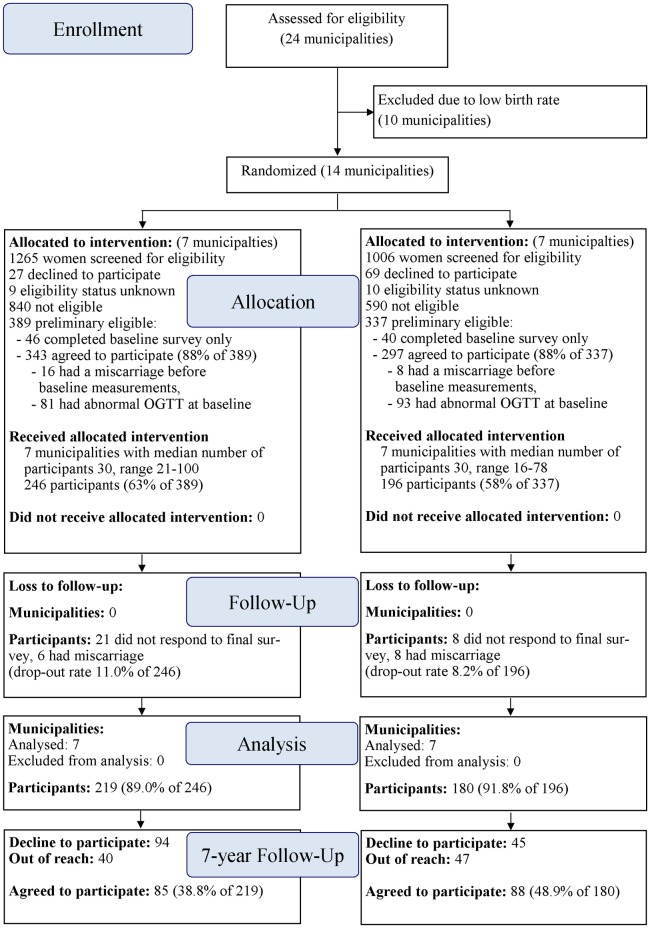
Flow diagram of the cluster-randomized trial from enrollment till 7 year follow-up.

A total of 173 (43.4%) participants of 399 original RCT were willing to participate the follow-up study. Included participants were almost equally from the original intervention (n = 85) and usual care group (n = 88) (Fig 1). The remainder 139 (34.8%) were not willing to participate and 87 (21.8%) were out of reach. Participant’s background information (age, education, smoking, number of children and working situation) did not differ between the intervention and usual care groups (Table 1).

**Table 1:** Characteristics (mean ± SD or frequency and percentage).

	Intervention group n = 85	Usual care group n = 88	p	Missing (Intervention/Control)
Age	37.7 (4.5)	38.1 (4.9)	0.66	-
Education level			0.23	2/0
Low	25 (30.1%)	25 (28.4%)		
Medium	28 (33.7%)	40 (45.5%)		
High	30 (36.1%)	23 (26.1%)		
Smoking			0.88	5/3
No	73 (91.3%)	77 (90.6%)		
Occasionally / daily	7 (8.7%)	8 (9.4%)		
Number of children			0.42	6/4
1	8 (10.1%)	12 (14.3%)		
≥2	71 (89.9%)	72 (85.7%)		
Work			0.68	5/3
Full- or part-time work	57 (71.3%)	63 (74.1%)		
Other (e.g. unemployed)	23 (28.7%)	22 (25.9%)		
Gestational diabetes during pregnancy	5 (6.3%)	4 (4.8%)		1

None of the women who were involved in the intervention or usual care group had T2DM or prediabetes or medication for T2DM or abnormal result in fasting blood glucose or 2-hour measurement in OGTT (Table 2). Additionally, glycosylated hemoglobin (HbA1c) was similar between the groups and all values were normal (Table 2). BMI was non-significantly lower in the intervention group as compared to the usual care group (27.3 vs. 28.1, p = 0.33). Waist circumference was almost the same in both groups (89.9 cm vs. 90.9 cm, p = 0.63).

**Table 2:** Outcome measures for effectiveness and cost-effectiveness during gestation and 7-year follow-up.

Outcomes	8–12 week gestation			7 year follow up			Change from 8–12 week to 7 year follow up			
	Mean (SD)		p-value^1^	Mean (SD)		p-value^1^	Mean (SD)		p-value^1^^,^^2^	Missing
	InterventionGroup (n = 85)	Usual Care Group (n = 88)	Difference between groups	Intervention Group (n = 85)	Usual Care Group (n = 88)	Difference between groups	Intervention Group (n = 85)	Usual Care Group (n = 88)	Difference between groups	(Intervention/Control)
***Mother***										
**Fasting blood glucose**	4.90 (0.22)	4.79 (0.29)	0.01	5.15 (0.48)	5.20 (0.42)	0.57	0.25 (0.49)	0.41 (0.39)	0.15	16/22
**Glucose levels in**										
1-h OGTT	6.61 (1.59)	6.15 (1.39)	0.09	6.24 (1.94)	5.99 (1.53)	0.43	-0.37 (1.96)	-0.16 (1.59)	0.91	21/26
2-h OGTT	5.32 (1.04)	5.18 (0.82)	0.39	5.65 (1.40)	5.33 (0.99)	0.14	0.33 (1.58)	0.15 (0.94)	0.20	21/26
**HbA1c**				34.6 (2.33)	34.8 (2.43)	0.76				15/12
**Insulin**	11.8 (6.94)	12.1 (8.02)	0.61	8.45 (4.25)	9.67 (6.67)	0.50	-3.39 (6.91)	-2.46 (9.72)	0.91	21/10
**Anthropometry**										
Weight (kg)	71.4 (16.2)	73.3 (11.0)	0.39	75.6 (17.3)	76.9 (13.1)	0.56	4.13 (6.56)	3.67 (7.59)	0.69	2/1
Body mass index	25.8 (4.95)	26.7 (4.13)	0.22	27.3 (5.48)	28.1 (5.08)	0.33	1.50 (2.41)	1.42 (3.09)	0.88	3/1
Waist circumference (cm)				89.9 (13.0)	90.9 (12.8)	0.63				11/13
**Physical activity**										
At least moderate (minutes/day)				39 (0.28)	37 (0.33)	0.72				14/15
Total (h+ minutes/day)				4.41 (1.30)	4.54 (1.12)	0.30				14/15
**Quality of life (15D)**	0.95 (0.04)	0.95 (0.05)	0.61	0.94 (0.06)	0.93 (0.06)	0.30	-0.01 (0.06)	-0.02 (0.05)	0.13	5/3
***Child***										
**Body mass index**				21.3 (3.79)	22.5 (4.31)	0.07				11/19
**HbA1c**				34.4 (2.04)	34.8 (2.32)	0.31				32/36
**Fasting blood glucose**				4.94 (0.49)	4.93 (0.41)	0.81				32/37

^1^ Linear regression models

^2^ Adjusted for baseline (8–12 week gestation)

Objectively measured moderate-to-vigorous physical activity time at seven-year follow-up was on average 38 minutes per day in both groups (Table 2). Objectively measured total physical activity (i.e., light, moderate and vigorous physical activity), did not differ between the groups either (4.41 hours vs. 4.54 hours, p = 0.30; Table 2). Respectively quality of life score (on 0–1 scale) at follow-up did not differ between the groups (0.94 vs. 0.93, p = 0.30; Table 2). Like in adults, children’s BMI at follow-up was on average lower among those whose mother attended at intensified physical activity and dietary counseling intervention (21.3 vs. 22.5, p = 0.07; Table 2) in which also age was taken into account. Glucose tolerance measurements (fasting blood glucose and glycosylated hemoglobin) of children were similar in both groups.

### Costs

The mean total cost per person was 30.6% lower in the intervention group than in the usual care group (€2,944 vs. €4,243; p = 0.74; Table 3). Absence from work was the highest separate cost in both groups but did not differ significantly between the groups either (means €1,992 vs. €3,070 and medians €1,351 vs. €1,158; p = 0.93).When absence from work was analyzed separately according to reason there was no considerable difference between the groups in the absence from work because of children (4.0 days vs. 5.0 days, p = 0.83, not shown in table). Meanwhile, women who were in the intervention group had on average 6.3 absence days during the last year and usual care group 11.3 days respectively (p = 0.55) because of women’s own sickness (not shown in table).

**Table 3:** Annual mean health care costs (mean and SD) and productivity costs.

		Intervention group (*n* = 80)		Control group (*n* = 85)		
	Unit cost (EUR)	Number of units	Mean cost (EUR)	Number of units	Mean cost (EUR)	*p*-value
**Direct costs**						
Occupational health care doctor	78/ visit	1.5 (2.2)	113.5	1.7 (4.5)	131.4	0.76
Primary care doctor	117/ visit	0.9 (1.5)	105.6	1.3 (1.5)	148.7	0.013
Special health care doctor	310/ visit	0.6 (1.0)	185.8	0.9 (2.2)	284.6	0.86
Registered nurse in primary care	51/ visit	0.4 (1.5)	21.1	0.4 (1.2)	18.6	0.21
Public health nurse in occupational health	42/ visit	0.5 (0.9)	19.0	0.5 (0.9)	19.0	0.87
Public health nurse in maternity clinic	58/ visit	0.9 (2.9)	52.6	0.5 (1.7)	26.9	0.56
Public health nurse in child health clinic	53/ visit	0.6 (1.6)	32.0	0.4 (0.9)	21.0	0.95
Public health nurse in family planning clinic	51/ visit	0.03 (0.16)	1.3	0.12 (0.62)	6.1	0.065
Physiotherapist	60/ visit	0.8 (2.5)	45.1	0.5 (1.7)	31.0	0.66
Medication			268.2		238.6	0.17
Inpatient days in primary care	250/ day	0.06 (0.40)	15.6	-	0.3	0.73
Inpatient days in special health care	820/ day	0.11 (0.50)	92.3	0.31 (1.18)	246.8	0.12
**Total direct costs**			**952**		**1,173**	0.33
**Productivity costs**						
Absence from work (€3,534/month)	193/day	10.3 (18.3)	1,992	16.1 (32.7)	3,070	0.93
**Total costs**			**2,944**		**4,243**	0.74

### Cost-effectiveness

**Fig 2:**
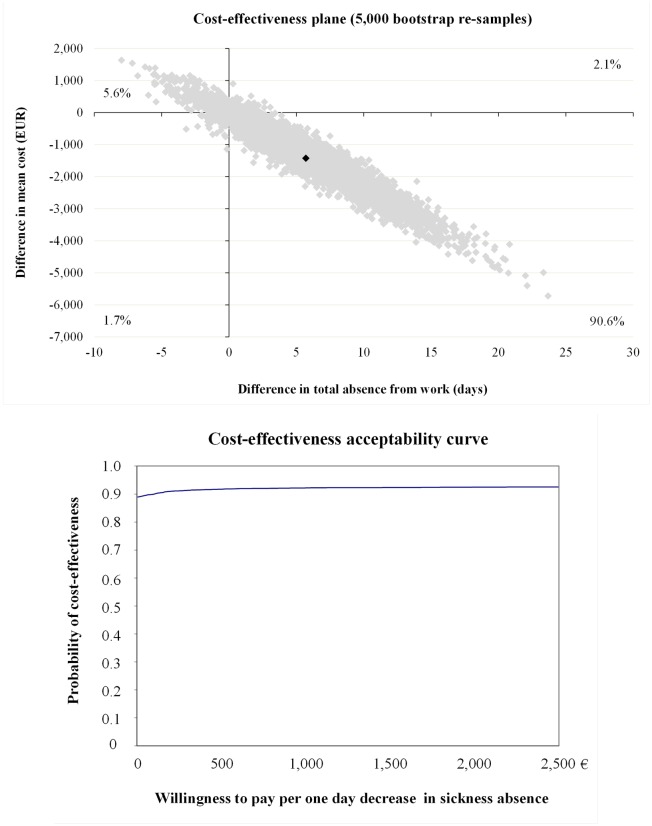
Cost-effectiveness plane and acceptability curve for absence from work.

**Fig 3:**
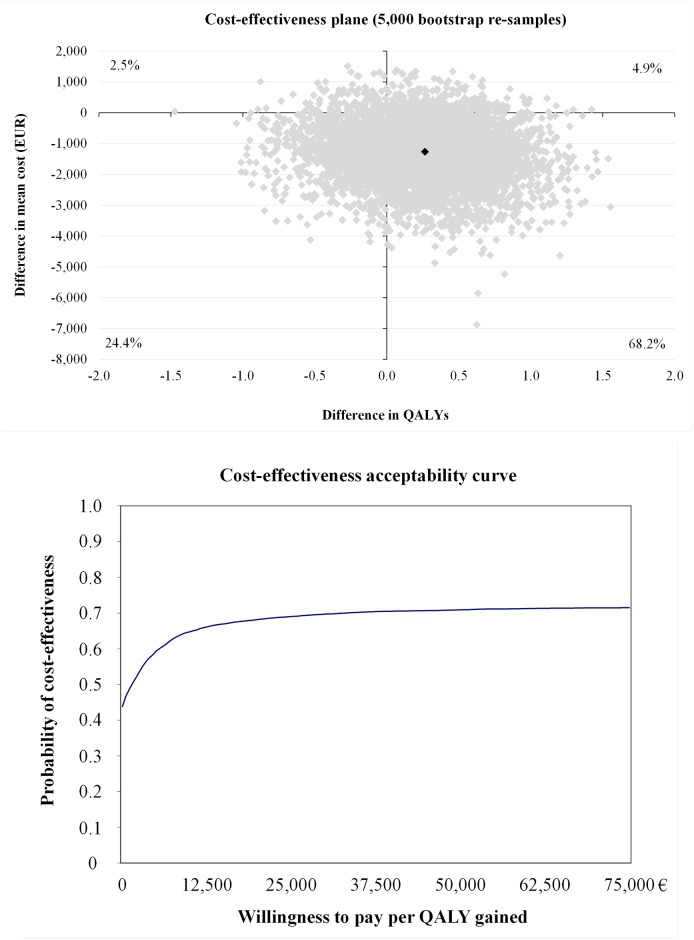
Cost-effectiveness plane and acceptability curve for QALY gained.

The incremental cost-effectiveness ratio (ICER) for absence from work was €-233 (Table 4). As the cost-effectiveness plane indicates (Fig 2), overall 90.6% of bootstrap pairs locate in the south-east quadrant indicating that the intervention after seven years of implementation was still cost-effective in terms of sickness absence. In other words, if society is willing to pay additional €100 per one avoided sickness absence day; there is a 90% probability of the intervention arm to be cost-effective (Fig 2). The ICER for QALY was -€5,386 (Table 4) with bootstrap 68.2% located in south-east quadrant (Fig 3). Intervention was not cost-effective for QALY gained because study indicated only 70% of probability of cost-effectiveness if society is willing to pay €33,000 per one-point improvement in QALY gained (Fig 3 and Table 4).

**Table 4:** Mean costs and effect differences (95% CI) between the intervention and usual care group and the sensitivity analysis, including incremental cost-effectiveness ratios and cost-effectiveness plane distributions.

	Sample size		Costs (EUR)	Effects	ICER	Distribution on CE plane (%)*			
	Intervention group	Control group	Δ of intervention—usual care (95% CI)	Δ of intervention—usual care (95% CI)		NE	SE	SW	NW
**Main analysis**									
Absence from work	80	83	-1,356 (-3,862 to 248)	5.82 (-1.26 to 15.7)	-233	2.1	90.6	1.7	5.6
QALYs	80	85	-1,300 (-3,600 to 224)	0.24 (-0.61 to 0.96)	-5,386	4.9	68.2	24.4	2.5
**Equal productivity costs**									
Absence from work	80	83	-233 (-978 to 306)	5.82 (-1.26 to 15.7)	-40	21.6	71.1	4.0	3.3
QALYs	80	85	-221 (-931 to 286)	0.24 (-0.61 to 0.96)	-917	17.3	55.8	19.9	7.0
**Complete case analysis**									
Absence from work	80	82	-1,394 (-4,002 to 97)	6.00 (-0.52 to 16.2)	-232	1.6	91.8	1.6	5.0
QALYs	80	82	-1,394 (-4,002 to 97)	0.20 (-0.65 to 0.91)	-6,949	4.2	65.8	27.6	2.4

* 95% CI: 95% confidence interval (‘bias-corrected and accelerated’ method), NE: north-east, SE: south-east, SW: south-west, NW: north-west.

#### Adherent group in original RCT

The adherent women (n = 24) had on average lower HbA1c value (33.7 vs. 34.8, p = 0.09; not shown in table) and higher quality of life (0.95 vs. 0.93, p = 0.09) compared to usual care group after seven years of intervention. Respectively children’s age perceived BMI was lower in adherent group (20.5 vs. 22.5, p = 0.04). There were no differences between adherent and usual care group in other variables.

### Sensitivity analysis

The first sensitivity analysis applied the assumption that the productivity costs were in the intervention group as high as in the usual care group and the second was a complete case analysis. The results of complete case analysis were quite similar to those in the basic analysis (Table 4).

## Discussion

Our follow-up study was not able to show effectiveness of gestational lifestyle counseling intervention on prevention of glucose intolerance or T2DM. In subgroup analysis children of the women adherent to the lifestyle aims had lower BMI as compared to children from usual care group mothers. Intervention was not cost-effective for QALY gained, but was cost-effective for sickness absence from work during last year.

Lack of effectiveness may be due to several reasons. First of all, it may be consequence of low intensity and similarities of the original counseling program as compared to the usual care group.

We have reported in our results from main trial [16] that usual care group participants received dietary counseling without specific aims or advices related to physical activity or gestational weight gain. Since diet is an important factor in prevention and treatment of diabetes, the differences in the groups may have been smaller than previously admitted. Overall effectiveness of the health behavior trials is rather low, based on previous reviews [12, 13, 36, 37].

Second reason for lack of effectiveness is found from low response rate of the study. More than one third of the women were reached but they were unwilling to participate oral glucose tolerance test or other measurements. Low response rate leads to low power, which cannot be denied in our results. It may also be possible that non-participating women had more adverse glucose intolerance profile and prevalent cases of T2DM, whereas the participating women were a selected healthy sample. Thus, healthy selection bias cannot be rejected when discussing the results of the study. On the other hand, proportion of drop-out women was equal in both groups in the follow-up study, thus increasing the reliability of the comparisons. This result also means that prevention of overt diabetes was successful in both groups, which can be considered as beneficial for all women participating to the original trial.

Thirdly, our results concerning the adherent group children’s BMI suggest that even small gestational changes in lifestyle may produce meaningful consequences to second generation. The finding of lower BMI among children may induce considerable cost savings later in life, because overweight increases the risk for T2DM and cardiovascular diseases later in life [3].

Although intervention was not cost-effective for QALY gained, cost-effectiveness for absence from work was a significant finding because the additional positive effect was gained at a very low price. Based at this study there is 90% probability that cost of €100 might shorten absence from work one day, which is half of workday’s cost to the society defined at this study (€193). From economical perspective, investment for more detailed and 2.5 hours longer lifestyle counseling [16] may save health care costs and decrease amount of sickness absence among women with risk of GDM. Procedures which increase physical activity and healthy nutrition and prevent excessive weight gain during pregnancy may save further health care costs because of mother’s and children’s lower BMI and decrease costs due to sickness absence.

On average 70% of total costs at this study were due to absence from work because of mothers own sickness or children’s sickness and 30% respectively were arisen because of use of health care services and medication. Since absence from work was the highest individual cost of all analyzed variables women should invest in their own work capacity, which may also improve women’s quality of life. However, reasons for lower sickness absence among intervention group remains unclear. According to Rasmussen et al. [38] high self-reported physical capacity was associated with lower long-term sickness absence among female health care workers. However, information on our study participant’s current physical capacity was not available.

One weakness of the study was that the follow-up study included only costs of the latest year; cost information was not available for longer time. However, we have no reason to believe that the latest year would have not been an average one. Cost-effectiveness of intervention was reported earlier [18] but cost data was not consistent with the latest year since the pregnancy.

Our study is the first follow-up study of a gestational lifestyle intervention, which included data both from mother and children. In addition, the advantage of the study was that sickness absences for less than ten days were included. Usually that information remains mainly at occupational health care and is not public information. Nearly half (48.6%) of the adult women are at least overweight (BMI ≥ 25 kg/m2) according to the latest reports [8]. Therefore, our findings may be generalized to the similar risk groups in other countries. There is a need for more long-term effectiveness and cost-effectiveness studies to examine findings related to GDM risk group.

### Conclusions

Pregnancy has both short- and long-term influences on health and susceptibility to future chronic disease both in mothers and their offspring. According to our findings, effectiveness of the lifestyle intervention was low, but none of the women who were involved in the follow-up study were diagnosed as having T2DM. Seven years after the intervention children’s BMI was lower in the intervention group of adherent mothers compared to usual care group. Long-term cost-effectiveness was found in absence from work but not in QALY gained. Procedures which increase physical activity and healthy nutrition and prevent excessive weight gain during pregnancy may save further health care costs because of children’s lower BMI and decrease costs due to sickness absence. There is a need for more long-term effectiveness and cost-effectiveness studies to examine whether findings with GDM risk group were equal with other postnatal women and their children.
